# Mitochondrial quality control in diabetes mellitus and complications: molecular mechanisms and therapeutic strategies

**DOI:** 10.1038/s41419-025-07936-y

**Published:** 2025-08-27

**Authors:** Yanling Chen, Xun Liu, Yixuan Liu, Yujia Li, Dingxiang Li, Zhigang Mei, Yihui Deng

**Affiliations:** 1 School of Integrated Chinese and Western Medicine, Hunan University of Chinese Medicine, Changsha, 410208 China; 2 Key Laboratory of Hunan Province for Integrated Traditional Chinese and Western Medicine on Prevention and Treatment of Cardio-Cerebral Diseases, Hunan University of Chinese Medicine, Changsha, 410208 China; 3 Loudi Central Hospital, Loudi, 417000 China; 4 School of Traditional Chinese Medicine, Hunan University of Chinese Medicine, Changsha, 410208 China

**Keywords:** Diabetes, Molecular biology

## Abstract

Diabetes mellitus (DM), a metabolic disease of globally health concern, is pathologically attributed to mitochondrial dysfunction, an essential component in disease progression. Mitochondrial quality control (MQC) acts as a critical defense mechanism for metabolic homeostasis, yet its implications in DM and its complications remain incompletely understood. This study thoroughly summarizes emerging evidence that delineates the molecular processes of MQC, with an emphasis on effector protein post-translational regulation, upstream signaling hubs, and interactions with other metabolic processes including ferroptosis and lipid metabolism. We highlight newly discovered processes involving mitochondrial-derived vesicles, licensed mitophagy, and mitocytosis that broaden the regulatory landscape of MQC, going beyond the traditionally recognized process including biogenesis, dynamics and mitophagy. MQC imbalance exacerbates insulin resistance, while impaired insulin signaling reciprocally compromises mitochondrial function, creating a vicious cycle of metabolic deterioration. Despite tissue-specific pathophysiology, diabetic complications exhibit identical MQC impairment including suppressed biogenesis, fission-fusion imbalance, and deficient mitophagy. Emerging therapies including clinical hypoglycemic agents and bioactive phytochemicals demonstrate therapeutic potential by restoring MQC. However, current strategies remain anchored to classical pathways, neglecting novel MQC mechanisms such as mitocytosis. Addressing this gap demands integration of cutting-edge MQC insights into drug discovery, particularly for compounds modulating upstream regulators. Future studies must prioritize mechanistic dissection of MQC novel targets and their translational relevance in halting metabolic collapse of diabetes progression. Since mitochondrial function is a cornerstone of metabolic restoration, synergizing precision MQC modulation with multi-target interventions, holds transformative potential for refine diabetic complications therapeutics.

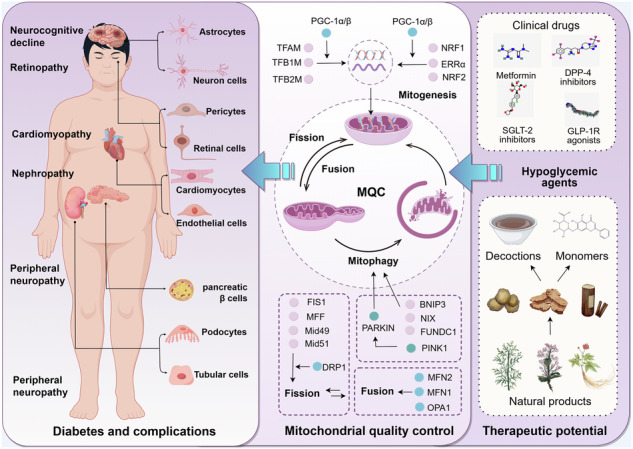

## Facts

- MQC governs insulin signaling and cellular maturation in metabolic tissues, maintaining metabolic homeostasis and function.
- Despite the tissue-specific manifestations, MQC impairment in diabetes accelerates systemic metabolic collapse with conserved pathogenic features including suppressed biogenesis and fusion, excessive fission, and deficient mitophagy.
- Emerging therapeutic strategies including clinical hypoglycemic agents and natural products exhibit great potential in the treatment of diabetes and complications by targeting MQC.

## Introduction

Diabetes mellitus (DM), a systemic metabolic disease characterized by chronic hyperglycemia and insulin resistance, poses a mounting global health burden, with a prevalence estimated to reach 783.2 million adults by 2045 [1]. Mitochondria are dynamic organelles essential for cellular energy metabolism [2, 3]. Metabolic stress including persistent hyperglycemia and dysregulated glucolipid metabolism could induce pancreatic mitochondrial dysfunction, especially in β cells, which is key to the onset and progression of diabetes, aggravating oxidative stress, inflammation, and glucolipotoxicity, driving diabetic multi-organ damage [4–8].

Mitochondrial quality control (MQC), a network encompassing biogenesis, dynamics (fission and fusion), and mitophagy, ensures mitochondrial integrity and metabolic flexibility [9]. Emerging evidence positions MQC as a linchpin in DM pathophysiology. MQC imbalance disrupts cellular ATP synthesis, amplifies oxidative damage, and perpetuates metabolic stress across insulin-sensitive tissues [10–13]. While the role of MQC in diabetes is well-documented previously, its tissue-specific regulation in diabetic complications, ranging from cardiac lipotoxicity to renal tubular injury, remains underexplored [14, 15].

This review synthesizes emerging insights into MQC mechanisms, elucidating their physiological and pathological interplay with DM. We posit that MQC imbalance serves as a co-pathogenic aspect of diabetic complications, and summarize emerging evidence of MQC molecular targets on diabetic complications. What is more, we discuss the therapeutic strategies targeting MQC to break the cycle of mitochondrial collapse and systemic metabolic decompensation. We searched PubMed for relevant English-language articles published up to April 2025 (final search date). The query terms related to MQC and diabetic complications: (“mitochondrial quality control” OR “mitochondrial biogenesis” OR “mitochondrial dynamics” OR “mitochondrial fission” OR “mitochondrial fusion” OR “mitophagy”) AND (“diabetes mellitus” OR “insulin resistance”) AND (“pancreas” OR “liver” OR “muscle” OR “adipose” OR “diabetic complications” OR “diabetic heart” OR “diabetic cardiomyopathy” OR “diabetic nephropathy” OR “diabetic kidney” OR “diabetic retinopathy” OR “diabetic retina” OR “diabetic neuropathy” OR “diabetic peripheral neuropathy” OR “diabetic brain”). Studies were included if they investigated mechanistic links between MQC and diabetes or its complications, provided molecular or therapeutic insights, and were peer-reviewed original research. Excluded articles included reviews, editorials, non-English publications, and studies lacking direct relevance to DM and MQC. Two independent reviewers screened titles and abstracts, followed by full-text assessment of eligible articles.

## Mitochondrial quality control

To ensure optimal function, mitochondria undergo a finely tuned quality control mechanism involving mitochondrial motility, mitochondrial-derived vesicles (MDVs), the ubiquitin-proteasome system, and cellular environmental factors [16].

### Mitogenesis

Mitogenesis replenishes mitochondrial mass via coordinated nuclear DNA (nDNA)- and mitochondrial DNA (mtDNA)-encoded programs. The peroxisome proliferator-activated receptor γ coactivator-1 (PGC-1) family, including PGC-1α, PGC-1β, and PRC, orchestrate this process by activating transcription factors NRF1, NRF2, and ERRα, as well as respiratory complex assembly [17–20]. While PGC-1α drives biogenesis in energy-demanding tissues including heart and muscle, PGC-1β compensates during brown adipogenesis [21]. PRC regulates NRF1/CREB-dependent transcription but is dispensable for basal biogenesis [22, 23]. Upstream regulators and post-translational modifications like PGC-1α monomethylation [24] fine-tune biogenesis, while mitochondrial preprotein translocases TOM and TIM complexes ensure nuclear-encoded protein import [25, 26]. Under stress, mitochondrial also transfer from neighboring cells and can rescue bioenergetic deficits [27, 28].

### Mitochondrial dynamics

Mitochondrial morphology adapts to metabolic demands via fission and fusion, governed by post-translational modifications (PTMs) of dynamin-related GTPases [29, 30]. Fusion merges outer (MFN1/MFN2) and inner (OPA1) membranes, enabling content exchange and cristae stabilization [31, 32]. Transient “kiss-and-run” fusion events allow selective matrix communication [33]. Fission, mediated by Drp1 recruitment to mitochondrial receptors (Mff, Mid49/51, Fis1), partitions damaged regions for degradation [34–38]. Imbalanced dynamics including excessive fission and defective fusion disrupt metabolic flexibility and amplify oxidative stress.

### Mitophagy

Mitophagy, a specialized form of cellular autophagy, selectively degrades damaged mitochondria via the Ser/Thr kinase PINK1/ the E3 ubiquitin ligase Parkin-dependent and -independent pathways. Depolarized mitochondria stabilize PINK1, which phosphorylates ubiquitin to recruit Parkin for OMM protein ubiquitination [39–41]. Parkin-independent pathways utilize OMM receptors including BCL2 and adenovirus E1B 19-kDa-interacting protein3 (BNIP3), BNIP3-like (BNIP3L/NIX), Bcl-2-like protein 13 (BCL2L13), FUN14 domain containing 1 (FUNDC1) and tacrolimus (FK506)-binding protein8 (FKBP8), as well as IMM phospholipids cardiolipin to recruit LC3-labeled autophagosomes [42, 43]. Regulatory crosstalk involves prohibitin 2 (PHB2), autophagy-beclin1-regulator1(AMBRA1), and Myosin VI (MYO6), which promote autophagosome-lysosome fusion [44–48].

### Novel horizons for MQC

Beyond canonical mitophagy, MQC encompasses adaptive mechanisms to preserve homeostasis under stress. MDVs act as a compensatory pathway when LC3-dependent autophagosome formation is impaired, selectively packaging misfolded proteins, oxidized lipids or damaged mtDNA into mitochondrial-derived compartments (MDCs) for lysosomal or peroxisomal degradation [49–53]. MDV biogenesis is regulated by microtubule-associated motor proteins MIRO1/2 and fission machinery components (Drp1, Mff, Mid49, Mid51), linking vesicle formation to mitochondrial dynamics [53].

Cells unable to recycle dysfunctional mitochondria intracellularly may transfer damaged components via extracellular vesicles for phagocytic clearance by neighboring cells (e.g., macrophages), a process termed “licensed mitophagy” [27, 28]. Recent work also identifies mitocytosis, a migrasome-mediated expulsion of mitochondria during cell migration. Under mild stress, motor proteins direct damaged mitochondria to migrasomes at the cell periphery, enabling extracellular disposal [54].

Furthermore, mitochondria-lysosome crosstalk enables localized repair through inner mitochondrial membrane-derived vesicles (VDIMs). Cristae damage triggers ROS-dependent activation of lysosomal TRPML1 channels, promoting VDAC1 oligomerization and herniation pore formation on the OMM. This facilitates extrusion of damaged IMM components, which are engulfed by lysosomes as VDIMs [55].

**Fig. 1:**
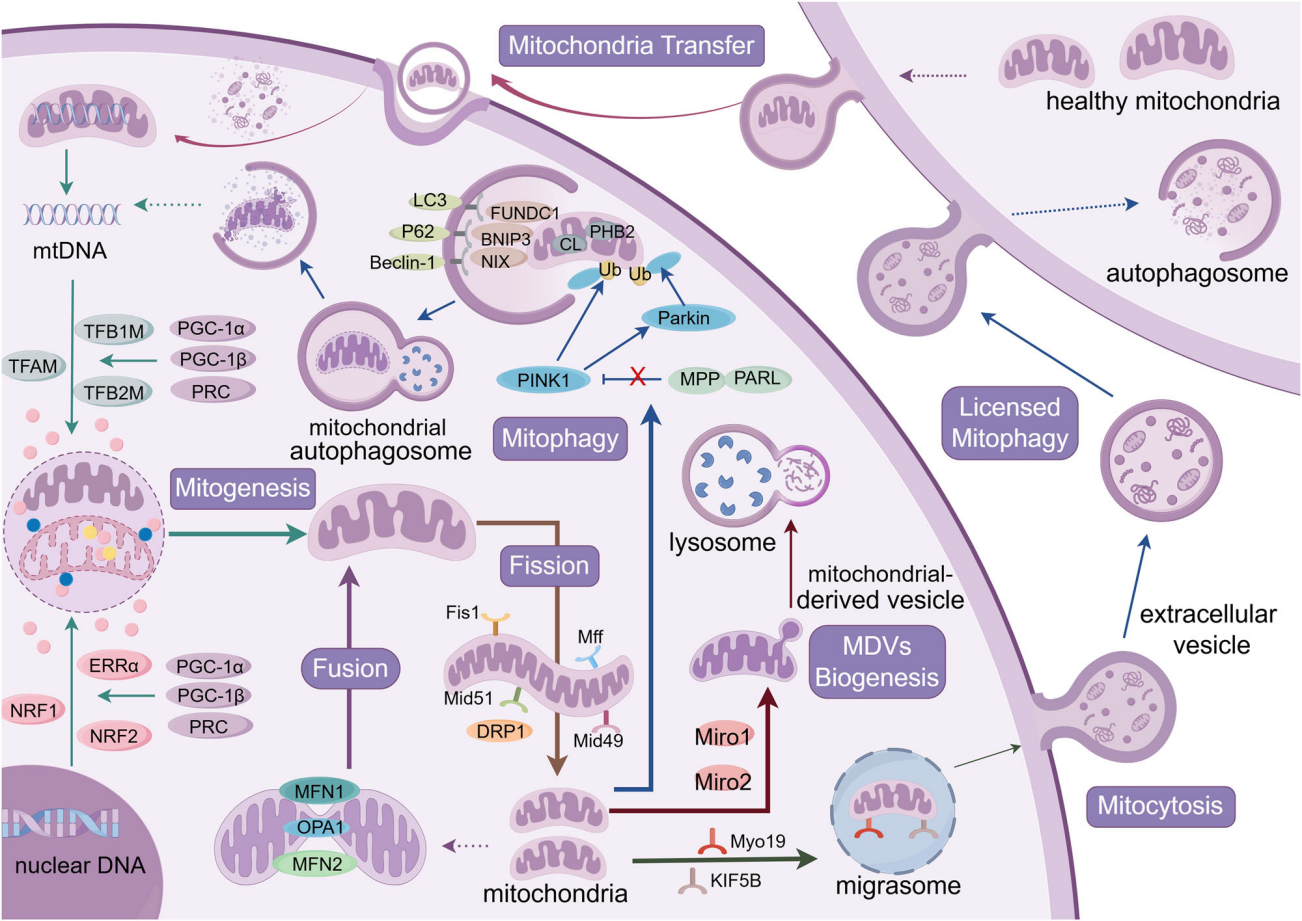
MQC mechanisms (Created with FigDraw). **Mitogenesis**: Nuclear DNA and mitochondrial DNA are responsible for the synthesis of new mitochondria, a process regulated by coactivators and transcription factors through membrane protein transport mechanisms. **Mitochondrial dynamics**: The fusion is mediated by OMM proteins (MFN1 and MFN2), and the fission is mediated by the interaction between DRP1 and fission factors (Fis1. Mff, Mid49, Mid51). **Mitophagy**: The process is mainly mediated by the PINK1/Parkin pathway and mitochondrial membrane protein receptor-dependent signaling pathway. **MDVs biogenesis**: Mitochondria selectively release components and even damaged mtDNA through MDVs, which are degraded through specific transport pathways to lysosomes. **Mitocytosis**: Mitochondria are translocated to migrasomes, and migrating cells selectively bind damaged mitochondria to motor proteins, localize them to the cell’s periphery, and ultimately discharge them outside the cell to be removed. **Licensed mitophagy**: A cell transfers its own damaged mitochondrial components to extracellular vesicles to be released and eliminated with the help of mitophagy of another cell. **Mitochondria transfer:** When endogenous mitogenesis is deficient, the cell replenishes exogenous healthy mitochondria from outside other cells via mitochondrial transfer.

These emerging pathways underscore MQC’s plasticity, offering novel therapeutic entry points to disrupt the cycle of mitochondrial dysfunction and metabolic disease (Fig. 1).

## The role of mitochondria in pancreatic beta cells

Mitochondria regulate pancreatic β-cell insulin secretion and peripheral insulin sensitivity through redox balance, metabolic flux, and interorganellar communication [56, 57]. Maintaining mitochondria turnover ensures establishment of sufficient mitochondria OXPHOS for islet β cells differentiation and maturation [58]. The mitochondrial tRNA-derived fragment interacts with electron transfer system complexes and paly pivotal role in mitochondrial oxidative phosphorylation and its coupling to insulin secretion of pancreatic β-cells [59].

In pancreatic β cells from type 2 diabetic subjects, the impaired secretory response to glucose is associated with a marked alteration of mitochondrial function and morphology. In particular, uncoupling protein-2 expression is increased, which leads to lower ATP, decreased ATP/ADP ratio, with consequent reduction of insulin release [60]. Hyperglycemic environment induces metabolic changes in β cells that significantly reduce mitochondrial metabolism and ATP synthesis, which contribute to the progressive failure of β-cells to respond to glucose in diabetes [61]. Inactivation of transcriptional factor YY1 impairs mitochondrial oxidative phosphorylation and mitochondrial function, resulting in the reduction of insulin secretion, β-cell mass, and glucose tolerance [62]. Lipotoxicity including saturated free fatty acids (FFAs) accumulation alters β-cell signal transduction [63], induce mitochondrial fragmentation and production of ROS in β cells, thereby promoting β-cell dysfunction and pancreatic insulin sensitivity changes [64–66]. Effective mitochondrial adaptive responses including mitochondrial redox signaling and mild uncoupling reduce β-cell oxidative stress and dysfunction under metabolic stresses like hypertriglyceridemia [67].

## MQC in the pathophysiology of insulin resistance and diabetes

Insulin resistance (IR), a hallmark of type 2 diabetes mellitus (T2DM), arises from impaired cellular responsiveness to insulin, disrupting glucose homeostasis and driving hyperglycemia. Both insulin insufficiency and IR converge on mitochondrial dysfunction, where defective MQC exacerbates metabolic dysregulation. Targeting MQC pathways offers a strategic avenue to uncouple mitochondrial failure from disease progression.

### MQC-insulin signaling crosstalk

Enhanced mitochondrial biogenesis improves ATP synthesis, facilitating insulin-stimulated glucose uptake via GLUT4 translocation [68]. Mitochondrial dynamics proteins directly modulate insulin signaling: Mfn2 stabilizes insulin receptor substrate 1 (IRS1) and coordinates mitochondrial-ER crosstalk [69], while phosphorylation of mitochondrial fission factor (MFFS131) promotes hepatic insulin sensitivity by tuning mitochondrial fragmentation [30]. Conversely, insulin regulates MQC via Akt/mTOR/FOXO1 signaling, suppressing Drp1/Fis1-mediated fission and enhancing PGC-1α-driven biogenesis [70–72]. Disruption of this bidirectional crosstalk which is evidenced by mitophagy-deficient β-cells with impaired insulin secretion [73, 74] fuels a self-perpetuating cycle of IR and mitochondrial collapse [75].

### MQC dysregulation in metabolic stress

Chronic hyperglycemia and lipid overload impair mitochondrial oxidative phosphorylation, elevating ROS production and depleting ATP synthesis [76]. ROS inactivate IRS1/2 via serine phosphorylation, exacerbating β-cell apoptosis and peripheral IR [77]. Downregulation of PGC-1α and NRF1 in T2DM reduces mitochondrial biogenesis, fatty acid oxidation, and respiratory capacity [78–80], while hyperglycemia-driven the upregulation of fission proteins fragments mitochondria, further amplifying oxidative stress [81–83]. Restoring deficient fusion or inhibiting excessive fission rescues IRS1/Akt signaling and glucose uptake, underscoring MQC’s role in metabolic flexibility [84, 85].

Defective mitophagy exacerbates IR by accumulating damaged mitochondria, triggering NLRP3 inflammasome activation and adipose inflammation [13, 86]. FUNDC1 knockout mice exhibit worsened obesity and IR under high-fat diets, linking impaired mitophagy to adipose metabolic dysfunction [87]. These findings position MQC as a nodal regulator of inflammation-metabolism crosstalk in diabetes.

## Impaired MQC in the pathophysiology of metabolic tissues associated with IR

MQC dysregulation induces a retrograde signaling program that impairs identity and maturity in insulin-sensitive tissues including pancreas, liver and adipose, yielding metabolic disorders and driving systemic IR and diabetes progression [88]. Restoring MQC plasticity in these tissues offers a strategic avenue to mitigate metabolic dysfunction (Table 1).

**Table 1:** Summary of MQC-targeted molecules in IR-associated metabolic tissues.

Tissues	In vivo	In vitro	Molecule targets	MQC mechanisms	Reference
Pancreas	HFD-fed mice	Non-diabetic human pancreatic sections; neonatal mouse beta cells	NRF2↓	Inhibiting mitogenesis	[95]
	/	Primary nondiabetic mouse islets	OGT ↓ , pdx1↓	Inhibiting mitogenesis	[96]
	Db/db mice	Mouse insulinoma βTC6 cells	HuD ↓ , MFN2↓	Inhibiting fusion	[97]
	/	Primary mouse islets with PA	TRPM2 ↑ , DRP1↑	Promoting fission	[98]
	/	Rat insulinoma INS-IE cells	hIAPP↑	Promoting fission, inhibiting mitophagy	[100]
	/	INS-1 cells	PPP3 ↓ , TFEB↓	Inhibiting mitophagy	[101]
Liver	HFD-fed mice	Primary mouse embryonic fibroblast; HeLa cells with PA	CerS6 ↑ , Mff↑	Promoting fission	[34]
	/	Primary mouse hepatocytes	AKT ↑ , MFFS131↑	Promoting fission	[30]
Skeletal Muscle	HFD/STZ-induced mice	Mouse C2C12 myoblast cells	miR-30d-5p ↑ , SIRT1 ↓ , PGC-1α ↓	Inhibiting mitogenesis	[106]
	HFD-fed SD rats	Mouse C2C12 and L6 cell lines, human skeletal myoblasts with PA	YWHAB/14-3-3β ↓ , BNIP3L ↑ , DNM1L↑	Promoting fission and mitophagy	[107]
	STZ-induced SAM-P8 and SAM-R1 mice	Mouse C2C12 myoblast cells with AGEs	MFG-E8 ↑ , HSPA1L ↓ , Parkin↓	Inhibiting mitophagy	[108]
Adipose Tissue	Crossing NuTRAP mice with Ucp1-Cre mice	Primary mouse WAT cells	JMJD1A ↓ , PGC-1a/b↓	Inhibiting mitogenesis	[110]
	HFD-fed mice	Primary mouse adipocytes, human primary preadipocytes, 3T3-L1 adipocytes, Lenti-X 293 T cells	RalA ↑ , Drp1↑	Promoting fission	[111]
	HFD-fed mice	HEK293T cells, immortalized human brown preadipocytes	TMEM135 ↑ , AKAP1 ↑ , Drp1↑	Promoting fission	[112]
	/	3T3-L1 preadipocytes, neonatal brown preadipocytes	Sirt5 ↓ , UCP1↓	Inhibiting mitophagy	[113]

*HFD* high-fat diet, *db/db mice* leptin receptor-deficient mice, *SAM-P8* senescence-accelerated mouse prone 8, *SAM-R1* senescence-accelerated mouse resistant 1, *PA* palmitic acid, *STZ* streptozotocin, *PA* palmitic acid, *SD* Sprague Dawley, *AGEs* advanced glycation end products, *HEK293T* human embryonic kidney 293 T.

### Pancreas

The pancreatic endocrine β-cells, central to systemic glucose homeostasis [89], are critically vulnerable to mitochondrial dysfunction in diabetes. The disruption of mitochondrial turnover and function in islets contribute to chronic exposure of pancreatic β-cell to high concentrations of FFAs, aggravating lipotoxicity-mediated suppression of glucose-stimulated insulin secretion [90–92]. Emerging evidence highlights that MQC failure in islets drives β-cell dysfunction through impaired glucose tolerance and exacerbated insulin resistance, accelerating diabetes progression [93, 94]. Notably, NRF2 deficiency under metabolic stress disrupts mitogenesis, triggering a marked decline in functional β-cell mass and systemic glucose dysregulation [95]. Concurrently, O-GlcNAc transferase (OGT) sustains β-cell physiology by enhancing pancreatic and duodenal homeobox 1 (Pdx1)-dependent mitogenesis, thereby preserving insulin secretion [96]. Dysregulated mitochondrial dynamics directly underpins β-cell death. For instance, RNA binding protein HuD overexpression rescues diabetic β-cell dysfunction by restoring MFN2-mediated mitochondrial fusion, whereas palmitate (a C16-FFA)- induced activation of TRPM2 elevates mitochondrial Zn²⁺, potentiating DRP1-driven mitochondrial fission, β-cell apoptosis, and insulin secretory failure [97, 98]. The Activation of LRH-1/NR5A2 targeting mitochondrial dynamics attenuates the autoimmune attack coupled to beta cell regeneration and islet survival in type 1 diabetes mellitus (T1DM) [99]. Furthermore, human islet amyloid polypeptide (hIAPP) aggregation in diabetes exacerbates mitochondrial fragmentation and suppresses mitophagy, leading to ROS overproduction and irreversible β-cell damage [100]. Disruption of the PPP3/calcineurin/TFEB axis impairs mitophagy, culminating in β-cell dysfunction and glucose intolerance [101]. Under glucotoxic conditions, GRP78 upregulation cause depletion of the antioxidant pool and promote mitophagy in pancreatic cells in T1DM [102]. Collectively, MQC impairment contributes to β-cell demise in diabetes, offering potential targets for interventions to preserve pancreatic β-cell mass and function.

### Liver

Hepatic MQC directly modulates glucose and lipid homeostasis. Activation of the AMPK/SIRT1/PGC-1α axis enhances mitogenesis, suppresses gluconeogenesis, and promotes fatty acid oxidation, reversing lipid accumulation and IR in T2DM [80, 103]. CerS6-derived sphingolipids, as a kind of hepatic lipid deposition, promote hepatic mitochondrial fragmentation and glucose disorders in a Mff-dependent manner, exacerbating obesity and IR [34]. Nutrient-sensing pathways further fine-tune mitochondrial dynamics. AKT-dependent phosphorylation of mitochondrial fission factor MFFS131 induces transient mitochondrial fragmentation, priming the liver for metabolic adaptation to feeding cycles [30]. Conversely, Drp1 and Fis1 upregulation disrupts oxidative phosphorylation, exacerbating hepatic steatosis and hyperglycemia [104]. As a catabolic process, mitophagy promotes mitochondrial fatty acid oxidation to inhibit hepatic fatty acid accumulation, thereby improving hepatic IR and metabolic syndrome [105].

### Skeletal muscle

MQC governs insulin-stimulated glucose uptake in skeletal muscle. Upregulation of miR-30d-5p undermines glycolipid metabolism in skeletal muscle by targeting SIRT1/PGC-1α-dependent mitogenesis [106]. Lipid infusion increases the expression of DRP1 and PINK1, regulating MQC networks to suppress insulin sensitivity in human skeletal muscle [75]. Nutrient storage stress triggers a BNIP3L-mediated signaling cascade that promotes mitochondrial fission and mitophagy, impairing myocyte glucose uptake with the activation of MTOR-RPS6KB and IRS1 phosphorylation [107]. In diabetic sarcopenia, MFG-E8 (lactadherin) is upregulated and induces mitophagy deficiency via the HSPA1L-Parkin pathway, thereby aggravating diabetic muscle atrophy [108]. Deubiquitinating enzymes including USP15 and USP30 activates DRP1-dependent mitochondrial fission, thereby regulating skeletal muscle MQC and insulin sensitivity in T2DM patients [109].

### Adipose tissue

Adipocyte MQC regulates systemic energy balance. JMJD1A, a histone demethylase, activates PGC-1-mediated mitogenesis and the formation of beige adipocytes in white adipose tissues (WAT), alleviating obesity and metabolic dysfunction [110]. In WAT, activation of small GTPase RalA promotes excessive mitochondrial fission, suppresses thermogenesis, and drives obesity-associated metabolic dysfunction [111]. TMEM135 overexpression promotes brown fat mitochondrial fission, counteracts obesity and insulin resistance, and rescues thermogenesis in adipose-specific peroxisome-deficiency [112]. FUNDC1-dependent mitophagy maintains adipose homeostasis, whereas FUNDC1 ablation in WAT disrupts mitochondrial integrity, exacerbates diet-induced obesity and IR [87]. Brown adipose tissue (BAT) relies on mitochondrial protein succinylation and malonylation to sustain thermogenic capacity, with defects in these post-translational modifications impairing mitophagy and glucose homeostasis in T2DM [86, 113].

## Impaired MQC in diabetic complications

Individuals with diabetes face a significantly elevated risk of macrovascular events, such as stroke and myocardial infarction, as well as microvascular complications, including retinopathy and nephropathy, compared to non-diabetic populations. Emerging evidence underscores MQC dysfunction as a pivotal contributor to diabetic vascular endothelial dysfunction. Hyperglycemia induces mitochondrial fragmentation, impaired fusion, and excessive fission and autophagy across various tissues, including the cardiovascular system, liver, and pancreas [114, 115]. FOXO1 inhibition mitigates high glucose (HG)-induced DRP1 overexpression and mitochondrial fission in endothelial cells, reducing reactive oxygen species (mtROS) overproduction and ameliorating vascular dysfunction [116]. Given that mtROS-driven endothelial injury underlies both macro- and microvascular diabetic complications [117, 118], targeting MQC represents a promising therapeutic avenue to attenuate disease progression [119].

### Diabetic cardiomyopathy

Diabetic cardiomyopathy (DCM), characterized by myocardial fibrosis and impaired cardiac function independent of coronary artery disease, is linked to MQC defects involving calcium dysregulation, oxidative stress, and lipid accumulation [120]. Cardiac mitochondrial damage and biogenesis are induced by oxidative stress in a chronic model of type 1 diabetes [121]. While compensatory mitophagy may protect against early lipid overload in high fat diet-induced DCM [122], persistent imbalances in mitochondrial dynamics which is marked by excessive fission and deficient fusion and mitophagy that drive DCM pathogenesis.

Recent research has identified novel targets for DCM that alleviate mitochondrial dysfunction and cellular apoptosis by modulating post-translational modifications (PTMs) of MQC proteins, including phosphorylation, acetylation, and methylation. PGAM5 dephosphorylates PHB2 at Ser^91^, disrupting MQC and exacerbating myocardial injury [123, 124]. Downregulated miR144 elevates RAC1, impairing AMPK/PGC-1α-mediated mitogenesis and promoting apoptosis [125]. CaSR enhances ubiquitination of MFN1, MFN2, and CX43 via GP78, disrupting mitochondrial fusion and energy metabolism [126]. PFKFB3 deficiency in diabetic hearts destabilizes OPA1, whereas NEDD4L-mediated OPA1 ubiquitination preserves mitochondrial integrity [127]. MAP4K4 disrupts GPX4-dependent redox balance, inducing Drp1 S-nitrosylation (SNO-Drp1), excessive fission, and ferroptosis [128]. MST1 inhibits SIRT3/PARKIN mitophagy and promotes DRP1 phosphorylation, accelerating mitochondrial fragmentation [129, 130]. By performing targeted metabolomics to characterize the metabolic phenotype of human myocardium of DCM, it is founded that RCAN1 upregulation activates DRP1 Ser^616^, driving lipid accumulation and metabolic remodeling in cardiomyocytes [131].

Epigenetic and epitranscriptomic mechanisms further modulate MQC in DCM. The NOTCH1/ALKBH5/YTHDF axis, regulated by m6A-mediated phase separation, suppresses NOTCH1 expression, promoting fission and fibrosis [132]. Similarly, BRD4 binds H3K27ac at the PINK1 promoter, repressing PINK1/Parkin mitophagy; BRD4 inhibition restores mitophagy and alleviates DCM [133].

MQC dysregulation intersects with lipid metabolism, calcium signaling, and ferroptosis in DCM. SFRP2, an adipokine, modulates mitochondrial dynamics via AMPK/PGC-1α and activates calcineurin/TFEB-dependent mitophagy [134, 135]. ORAI1-mediated Ca^2+^ influx activates DRP1, exacerbating fission and hypertrophy. Targeting ORAI1/p-ERK1/2/CnA normalizes calcium homeostasis [136]. DUSP26 rescues mitochondrial dynamics and lipid metabolism via FAK/ERK signaling [137]. LGR6 deficiency impairs STAT3/PGC-1α, aggravating ferroptosis and biogenesis defects [138].

Key transcription factors integrate MQC with metabolic stress. STAT3 activation by BNP sustains OPA1-mediated fusion, while FOXO1 competes with STAT3 for 14-3-3 binding, impairing mitochondrial homeostasis under hyperglycemia [139, 140]. PPARα downregulation reduces MFN2 expression, contributing to dynamics imbalance [141]. ALDH2 preserves mitochondrial function via Akt/GSK3β/Foxo3a signaling and PARKIN-dependent mitophagy [142].

DCM pathogenesis involves a complex interplay of MQC defects, PTMs, and transcriptional and epigenetic dysregulation. Targeting nodes such as DRP1 phosphorylation, mitophagy pathways, or redox balance offers therapeutic potential. Future studies should clarify the spatiotemporal dynamics of MQC disruption and optimize strategies to restore mitochondrial homeostasis in diabetes. (Table 2)

**Table 2:** Summary of MQC-targeted molecules in diabetic cardiomyopathy.

In vivo	In vitro	Molecule targets	MQC mechanisms	Reference
HFD-fed mice	HFD-fed mice cardiomyocytes	**ATG7** ↓ , PARKIN↓	Inhibiting mitophagy	[122]
STZ-induced mice	Primary NMCMs	**PGAM5** ↑ , PHB2 ↓ , DRP1 ↑ , FIS1 ↑ , MFN1 ↓ , MFN2 ↓ , PARKIN ↓ , Beclin1 ↓ , ATG5 ↓ , PGC1α ↓ , NRF2 ↓ , TFAM↓	Promoting fission, inhibiting mitogenesis, fusion and mitophagy	[133, 134]
STZ-induced mice	Primary NRCMs and H9c2 cells with HG	**miR-144** ↓ **, RAC-1** ↑ **, AMPK** ↓ , PGC-1α ↓ , NRF1 ↓ , TFAM↓	Inhibiting mitogenesis	[125]
/	Primary NRCMs with HG	**CaSR** ↓ , GP78 ↑ , MFN1 ↓ , MFN2 ↓ , CX43 ↓ , FIS1 ↑ , DRP1↑	Inhibiting fusion, promoting fission	[126]
Db/db mice	/	**PFKFB3** ↓ , NEDD4L, OPA1↓	Promoting fission	[127]
Db/db mice	Primary HCMECs with HG	**MAP4K4** ↑ , GPX4 ↓ , SNO-DRP1 ↑ , p-Drp1^S616^ ↑ , p-Drp1^S637^ ↓	Promoting fission and ferroptosis	[128]
HFD/STZ-induced mice	Primary NMCMs with HG	**MST1** ↑ , SIRT3 ↓ , DRP1 ↑ , p-Drp1^S616^ ↑ , p-Drp1^S637^ ↓ , PARKIN↓	Promoting fission, inhibiting mitophagy	[129, 130]
Db/db mice	H9c2 cells with HG and PA	**RCAN1** ↑ , p-Drp1^S616^ ↑	Promoting fission	[131]
HFD/STZ-induced mice; db/db mice	Primary neonatal mouse cardiac fibroblasts with HG and HF	**ALKBH5** ↓ **, YTHDF2** ↑ , NOTCH1 ↓ , ZEB1 ↓ , DRP1↑	Promoting fission	[132]
HFD-fed mice	NMCMs with PA	**BRD4** ↑ , PINK1 ↓ , PARKIN ↓ , TIM23 ↑ , VDAC1↑	Inhibiting mitophagy	[133]
HFD/STZ-induced SD rats	H9c2 cells with HG and PA	**SFRP2** ↓ , AMPK ↓ , FZD5 ↓ , TFEB ↓ , PGC-1α ↓ , NRF1 ↓ , TFAM ↓ , DRP1 ↑ , FIS1 ↑ , MFN1 ↓ , MFN2 ↓ , PARKIN ↓ , FUNDC1 ↓ , NIX ↓ , BNIP3 ↓ ,	Promoting fission, inhibiting mitogenesis, fusion, and mitophagy	[134, 135]
HFD-fed ZDF rats; db/db mice	Primary NRCMs with HG	**ORAI1** ↑ , CnA ↑ , p-ERK1/2 ↑ , DRP1 ↑ , p-Drp1^S616^ ↑ , p-Drp1^S637^ ↓ , OPA1 ↓ , MFN2↓	Promoting fission, inhibiting fusion	[136]
db/db mice; STZ-induced mice	Primary NRVMs, H9c2 and AC16 cardiomyocytes with HG and PA	**DUSP26** ↓ , FAK, ERK, DRP1 ↑ , MFN1 ↓ , MFN2 ↓ , OPA1↓	Promoting fission, inhibiting fusion	[137]
HFD/STZ-induced mice	HL1 cardiomyocytes with HG	**LGR6** ↓ , p-STAT3 ↓ , PGC-1α ↓ , cTNI ↑ , MDA ↑ , PTGS2↑	Reducing mitogenesis and mitochondrial respiratory capacity, promoting ferroptosis	[138]
STZ-induced mice	Primary mouse cardiomyocytes with HG	**BNP** ↓ , PKG ↓ , STAT3 ↓ , OPA1↓	Inhibiting mitochondrial fusion	[139]
HFD/STZ-induced SD rats; db/db mice	Primary NMCMs and H9c2 cells with HG	**FOXO1** ↑ , STAT3 ↓ , DRP1 ↑ , MFN1 ↓ , MFN2 ↓ , OPA1 ↓ , PINK1 ↓ , PARKIN↓	Promoting fission, inhibiting fusion, and mitophagy	[140]
Db/db mice	Primary NRCMs with HG, PA and OA	**PPARα** ↓ , MFN2↓	Promoting fission	[141]
STZ-induced mice	H9c2 cells with HG	**ALDH2** ↓ , Akt ↓ , GSK3β ↓ , FOXO3a ↓ , PARKIN↓	Inhibiting mitophagy	[142]

*HF* high fat, *HFD* high-fat diet, *db/db mice* leptin receptor-deficient mice, *HG* high glucose, *STZ* streptozotocin, *PA* palmitic acid, *OA* oleate acid, *SD* Sprague Dawley, *ZDF* Zucker diabetic fat, *SNO-Drp1* S-nitrosylation of Drp1, *HCMECs* human cardiac microvascular endothelial cells, *NRCMs* neonatal rat cardiomyocytes, *NRVMs* neonatal rat ventricular myocytes, *NMCMs* neonatal mouse cardiomyocytes.

### Diabetic nephropathy

Diabetic kidney disease (DKD), a chronic complication driven by persistent hyperglycemia and glomerular hyperfiltration, remains a leading cause of end-stage renal disease globally [143]. Pathologically, DKD is marked by altered glomerular filtration rates, proteinuria, and dysregulation of metabolites and ions. Podocytes, essential components of the glomerular filtration barrier, are particularly vulnerable to hyperglycemia-induced damage, contributing to mitochondrial dysfunction and disease progression. Emerging evidence underscores the centrality of mitochondrial homeostasis in maintaining renal health under diabetic conditions [144].

Mitogenesis is critically regulated in DKD through metabolic and signaling pathways. Hyperglycemia suppresses pyruvate kinase M2 (PKM2) tetramerization and activity via sulfenylation in podocytes, impairing glycolytic flux and amplifying toxic glucose metabolite accumulation. Restoring PKM2 activity enhances glucose metabolism, upregulates PGC-1α expression, and promotes mitochondrial biogenesis, thereby mitigating renal injury [145]. Conversely, overexpression of p66SHC, a redox-sensitive adaptor protein, suppresses mitogenesis by downregulating SIRT1, PGC-1α, NRF1, and TFAM, exacerbating mitochondrial ROS production and podocyte damage [146]. Similarly, mitochondrial glycerol 3-phosphate dehydrogenase (mGPDH) deficiency in diabetic models disrupts PGC-1α-dependent mitogenesis, activating the RAGE-S100A10 axis to drive oxidative stress and renal injury [147]. Additional regulators include AKAP1, which phosphorylates Larp1 via PKC signaling to inhibit TFAM-mediated mtDNA replication, and adiponectin receptor 1 (AdipoR1), whose deficiency impairs the CREB/PGC-1α/TFAM axis. Exogenous adiponectin supplementation rescues mitogenesis and alleviates tubular dysfunction, highlighting therapeutic potential [148, 149]. Progranulin (PGRN) depletion further disrupts mitochondrial homeostasis via Sirt1/PGC-1α/FOXO1 signaling, while the lncRNA PVT1 exacerbates fission and bioenergetic deficits by destabilizing AMPKα through TRIM56 interaction [150, 151]. Telomerase reverse transcriptase (TERT) also safeguards renal function by sustaining AMPK/PGC-1α-mediated mitochondrial energy balance [152].

Dysregulated mitochondrial dynamics, intertwined with hypoxia, lipid abnormalities, and calcium dyshomeostasis, further propagate DKD. Hypoxia-inducible factor-1α (HIF-1α) upregulation in tubular cells induces heme oxygenase-1 (HO-1) to stabilize mitochondrial dynamics and attenuate hypoxia-driven damage [153]. ALCAT1 expression is increased in the glomeruli of DKD patients, accompanying with significant mitochondrial damage [154]. Lipidomic perturbations, such as ALCAT1-mediated cardiolipin oxidation, impair mitophagy via AMPK signaling, while the ChREBP/GNPAT/plasmalogen axis links lipid metabolism to mitochondrial fission [154, 155]. RIPK3 overexpression exacerbates podocyte injury by activating PGAM5/Drp1-mediated fission through necroptosis, illustrating crosstalk between cell death pathways and organelle dynamics [156].

Electron transport chain (ETC) dysfunction exacerbates bioenergetic failure in DKD. NDUFS4 deficiency disrupts complex I integrity, impairing cristae morphology via STOML2 interaction, whereas IGF2BP3 stabilizes CAMK1 to inhibit fission and apoptosis through m6A modification [157, 158]. Calcium signaling further modulates mitochondrial fitness. CaMKKβ sustains AMPK phosphorylation to support function, but NEDD4L ubiquitination under hyperglycemia destabilizes CaMKKβ, promoting fission [159]. TRIM22 and WTAP form an m6A-dependent regulatory axis targeting OPA1 ubiquitination, thereby disrupting fusion and amplifying injury [160]. HGF maintaining mitochondrial homeostasis in type 1 diabetic podocytes via decreasing ARF6-dependent DRP1 translocation [161].

Impaired mitophagy, coupled with MAM disruption, ferroptosis, and oxidative stress, is a hallmark of DKD. KCa3.1 channel deficiency impedes TGF-β1/BNIP3-dependent mitophagy, while DsbA-L depletion activates JNK/MFF/DRP1 signaling to drive fission. Conversely, DsbA-L preserves MAM integrity via HELZ2/MFN2 to promote mitophagy, illustrating context-dependent roles [162–164]. TNFAIP8L1/TIPE1 exacerbates tubular injury by degrading the mitophagy receptor PHB2, whereas UHRF1 enhances PINK1-dependent mitophagy and suppresses ferroptosis via TXNIP inhibition [165, 166]. MFN2-SERCA2 interactions and VDR activation restore MAMs and mitophagy through the FUNDC1 pathway, while PACS-2 coordinates fission inhibition and BECN1-dependent mitophagosome formation [167, 168]. YAP1 inactivation disrupts both mitogenesis and PINK1/Parkin-mediated mitophagy, skewing macrophage polarization via CXCL1, whereas TRPC6/calpain-1 signaling impairs mitophagy to fuel inflammation [169, 170]. DLAT, downregulated in diabetes, enhances AMPK-driven mitophagy, offering protection against renal dysfunction [171]. N-Acetyl-Cysteine combined with insulin reverses glomerular wrinkling and fibrosis by regulating the mitochondrial dynamics and FUNDC1-mediated mitophagy in type 1 diabetic nephropathy canine [172].

These findings underscore the multifaceted interplay between mitochondrial homeostasis and diabetic renal pathology, with emerging therapeutic targets summarized in Table 3.

**Table 3:** Summary of MQC-targeted molecules in diabetic nephropathy.

In vivo	In vitro	Molecule targets	MQC mechanisms	Reference
STZ-induced mice	Primary mouse podocytes with HG	**PKM2** ↓ , PGC1α ↓	Inhibiting mitogenesis	[145]
HFD/STZ-induced SD rats	HK-2 cells with HG	**P66SHC** ↑ ; SIRT1 ↓ , PGC-1α ↓ , NRF1 ↓ , TFAM ↓ , MFN1 ↓ , DRP1↑	Inhibiting mitogenesis, promoting fission and apoptosis	[146]
STZ-induced mice; db/db mice	Mouse podocytes with HG	**mGPDH** ↓ , S100A10 ↑ , RAGE ↑ , PGC-1α ↓	Inhibiting mitogenesis, increasing mitochondrial ROS	[147]
STZ-induced SD rats; db/db mice	Human podocytes with HG	**AKAP1** ↑ , PKCβ ↑ , p-LARP1 ↑ , TFAM↓	Inhibiting mitogenesis, reducing mitochondrial DNA replication	[148]
HFD/STZ-induced SD rats	NRK-52E cells with HG	**AdipoR1** ↓ , CREB ↓ , PGC-1α ↓ , TFAM↓	Inhibiting mitogenesis	[149]
Human renal biopsies; STZ-induced mice	Human podocytes, rat glomerular endothelial cells, and rat glomerular mesangial cells with HG	**PGRN** ↓ , SIRT1 ↓ , PGC-1α ↓ , FOXO1 ↓ , PARKIN ↓ , PINK1↓	Inhibiting mitogenesis and mitophagy	[150]
Human renal biopsies; STZ-induced mice	Human podocytes, primary mouse renal podocytes with HG	**PVT1** ↑ , TRIM56 ↑ , AMPKα ↓ , PGC-1α ↓ , TFAM ↓ , DRP1↑	Inhibiting mitogenesis, promoting fission and inflammatory response	[151]
HFD/STZ-induced mice; db/db mice	HK-2 cells with HG and HF	**TERT** ↓ , AMPKα ↓ , PGC-1α ↓	Inhibiting mitogenesis	[152]
STZ-induced mice	Primary mouse proximal tubular epithelial cells; HK-2 cells with CoCl2	**HIF-1α** ↑ **, HO-1** ↓ , DRP1 ↑ , p-DRP1 ↑ , FIS1 ↑ , MFN1 ↓ , MFN2↓	Promoting fission, inhibiting fusion	[153]
Human diabetic renal biopsies; db/db mice	Human podocytes with HG	**ALCAT1** ↑ , AMPK ↓ , DRP1 ↑ , p-Drp1^S637^ ↓ , FIS1 ↑ , OPA1 ↓ , MFN2↓	Inhibiting fusion, promoting fission and apoptosis	[154]
Db/db mice	Primary mouse podocytes; HEK 293T cells with HG	**ChREBP** ↑ , GNPAT↑	Promoting fission	[155]
Human diabetic kidney biopsies; HFD-fed mice	Human and mouse podocytes with HG	**RIPK3** ↑ , MLKL ↑ , PGAM5 ↑ , Drp1 ↑ , p-Drp1^S616^ ↑	Promoting fission	[156]
Db/db mice	Primary mouse podocytes; HEK 293T cells with HG	**NDUFS4** ↓ , STOML2 ↓ , OPA1↓	Promoting fission	[157]
STZ-induced mice	HK-2 cells with HG	**IGF2BP3** ↓ , CAMK1 ↓ , DRP1 ↑ , p-DRP1 ↑ , FIS1 ↑ , MFN1 ↓ , MFN2↓	Promoting fission and apoptosis	[158]
Db/db mice	HK-2 cells with HG	**NEDD4L** ↑ , CaMKKβ ↓ , AMPK ↓ , p-DRP1 ↑ , MFN2↓	Promoting fission	[159]
Db/db mice	HK-2 cells with HG	**WTAP** ↑ , IGF2BP1 ↑ , TRIM22 ↑ , OPA1↓	Inhibiting fusion	[160]
Db/db mice	Immortalized podocytes with HG	**HGF** ↓ , ARF6 ↓ , DRP1↑	Promoting fission	[161]
STZ-induced mice	HK-2 cells with HG	**KCa3.1** ↑ , TGF-β1 ↑ , BNIP3 ↓ , DRP1 ↑ , OPA1↓	Promoting fission, inhibiting mitophagy and fusion	[162]
STZ-induced mice	HK-2 cells with HG	**DsbA-L** ↓ , JNK ↑ , HELZ2 ↓ , MFF ↑ , DRP1 ↑ , MFN2 ↓ , ATG14 ↓ , Beclin1↓	Promoting fission, inhibiting mitophagy	[163, [164]
STZ-induced mice	HK-2 cells with HG	**TIPE1** ↑ , PHB2↓	Inhibiting mitophagy	[165]
HFD/STZ-induced mice	HK-2 cells with HG	**UHRF1** ↓ , TXNIP ↑ , PINK1 ↓ , PARKIN↓	Inhibiting mitophagy, promoting ferroptosis	[166]
STZ-induced SD rats	HK-2 cells with HG and TGF-β	**VDR** ↓ , MFN2 ↓ , FUNDC1 ↓ , LC3II↓	Inhibiting mitophagy, promoting fission	[167]
STZ-induced mice	Primary mouse proximal tubular cells; HK-2 cells with HG	**PACS-2** ↓ , DRP1 ↑ , BECN1↓	Inhibiting mitophagy, promoting MAMs disruption	[168]
HFD/STZ-induced mice	HK-2 cells with HG and PA	**YAP1** ↓ , p-YAP1 ↑ , PGC-1α ↓ , TFAM ↓ , PINK1 ↓ , PARKIN↓	Inhibiting mitogenesis and mitophagy	[169]
STZ-induced mice	HK-2 cells with HG	**TRPC6** ↑ , Calpain-1 ↑ , PINK1 ↓ , PARKIN↓	Inhibiting mitophagy	[170]
HFD/STZ-induced mice; db/db mice	HK-2 cells with HG	**DLAT** ↓ , AMPK↓	Inhibiting mitophagy	[171]

*HF* high fat, *HFD* high-fat diet, *db/db mice* leptin receptor-deficient mice, *HG* high glucose, *STZ* streptozotocin, *PA* palmitic acid, *SD* Sprague Dawley, *HK-2* human kidney proximal tubular, *HEK* human embryonic kidney, *MAM* mitochondria-associated membranes.

### Diabetic retinopathy

Diabetic retinopathy (DR), a neurovascular complication of chronic hyperglycemia, is characterized by retinal glial network dysfunction, microvascular damage, and pathological neovascularization, culminating in vision loss and blindness [173]. The retina, composed of neurovascular units integrating endothelial cells, Müller glia, pericytes, ganglion cells, and pigment epithelial cells, undergoes mitochondrial dysregulation under hyperglycemic stress, marked by impaired mitophagy and aberrant mitochondrial dynamics. Emerging evidence highlights MQC as a pivotal therapeutic target to counteract metabolic memory and mitigate DR progression [174, 175].

Retinal vascular endothelial cells (RECs), critical for nutrient exchange and barrier integrity, exhibit disrupted mitochondrial homeostasis in diabetes. Hypermethylation of the MFN2 promoter, driven by Dnmt1 binding and diminished SP1 recruitment, suppresses MFN2 expression, while SIRT1 overexpression reduces MFN2 acetylation and GTPase activity, impairing mitochondrial fusion [176, 177]. Hyperglycemia further upregulates SENP3, which deSUMOylates Drp1 to promote fission, exacerbating blood-retinal barrier dysfunction [178]. Loss of PON2, glycated by N-carboxymethyl lysine (CML), amplifies ER stress, inflammation, and mitochondrial fragmentation, whereas TGR5 activation inhibits Drp1 via Ca^2+^-PKCδ signaling and enhances PINK1/Parkin-mediated mitophagy by displacing mitochondrial hexokinase 2 (HK2) [179, 180]. Methylglyoxal (MGO)-mediated glycation suppresses glyoxalase 1 (GLO1) and downregulates OPA1/MFN1, aggravating oxidative injury, while VDAC1 sustains mitophagy and restrains NLRP3 inflammasome activation in RECs [181, 182].

Pericytes, essential for vascular stability, succumb to hyperglycemia-induced mitochondrial fission via EPAC1-DRP1 signaling, driving ROS overproduction and microvascular damage [183]. In retinal pigment epithelial cells (RPECs), copper transporter 1 (CTR1) overexpression disrupts copper homeostasis, inducing oxidative stress and mitochondrial dysfunction, while TIN2 accumulation impairs PINK1-dependent mitophagy via mTOR, exacerbating cellular senescence and tight junction disruption [184, 185]. SIRT3, however, counteracts hyperglycemia by activating AMPK/mTOR/ULK1 and FOXO3a/PINK1/Parkin pathways, restoring mitophagy and attenuating RPEC apoptosis [186, 187].

Retinal ganglion cells (RGCs), vulnerable to oxidative stress, are protected by DJ-1, which scavenges ROS and restores mitochondrial homeostasis under hyperglycemic conditions [188]. Müller glia, pivotal for retinal repair, exhibit TXNIP-driven mitochondrial fission and Parkin-dependent mitophagy in response to hyperglycemia, alongside reactive gliosis marked by GFAP upregulation [189].

Collectively, these findings delineate mitochondrial dysregulation as a central axis in DR pathogenesis, with therapeutic strategies targeting MQC components offering promise to preserve retinal integrity (Table 4).

**Table 4:** Summary of MQC-targeted molecules in diabetic retinopathy.

In vivo	In vitro	Molecule targets	MQC mechanisms	Reference
STZ-induced rats	HRECs with HG	**DNMT1** ↑ , SP1 ↓ , SIRT1 ↓ , MFN2↓	Inhibiting mitochondrial fusion	[186, 187]
HFD-fed and STZ-induced mice	Primary mRMECs with HG	**SENP3** ↑ , DRP1**↑**	Inhibiting mitochondrial fission	[178]
/	HRECs with HG	CML ↑ **, PON2** ↓ , FIS1 ↑ , MFN2↓	Promoting mitochondrial fission	[179]
STZ-induced rats	HRECs with HG	**TGR5** ↓ , PKCδ ↑ , HK2 ↓ , DRP1 ↑ , PINK1 ↓ , PARKIN↓	Promoting mitochondrial fission, inhibiting mitophagy	[180]
/	Primary BRECs with HG	**MGO** ↑ , GLO1 ↓ , MFN1 ↓ , OPA1↓	Inhibiting mitochondrial fusion	[181]
/	HRCECs with HG	**VDAC1** ↓ , PINK1 ↓ , NLRP3**↑**	Inhibiting mitophagy	[182]
STZ-induced mice	Primary HRPCs with HG	**EPAC1** ↑ , DRP1**↑**	Promoting mitochondrial fission	[183]
/	Human RPECs with HG	**CTR1** ↑ , PGC1β ↓ , MFN2 ↓ , FIS1**↑**	Promoting mitochondrial fission, inhibiting mitogenesis and mitochondrial fusion	[184]
STZ-induced mice	Human RPECs with HG	**TIN2** ↑ , mTOR ↑ , SA-β-gal ↑ , PINK1 ↓ , PARKIN↓	Inhibiting mitophagy, promoting cell senescence	[185]
/	Human RPECs with HG	**SIRT3** ↓ , AMPK ↓ , mTOR ↑ , ULK1 ↓ , PINK1 ↓ , PARKIN↓	Inhibiting mitophagy	[186, [187]
STZ-induced rats	Rat retinal Müller glial cells with HG	**TXNIP** ↑ , GFAP ↑ , DRP1 ↑ , PARKIN**↑**	Promoting mitochondrial fission and mitophagy	[188]

*HRECs* human retinal vascular endothelial cells, *BRECs* bovine retinal endothelial cells, *HRCECs* human retinal capillary endothelial cells, *HRPCs* human retinal pericytes, *RPECs* retinal pigment epithelial cells, *mRMECs* murine retinal microvascular endothelial cells.

### Diabetic neuropathy

Diabetic neuropathy, encompassing central neurocognitive decline and diabetic peripheral neuropathy (DPN), represents a debilitating complication of chronic hyperglycemia, driven by glucotoxicity-induced neuronal and cerebrovascular dysfunction [190–192]. As mitochondria serve as the primary hubs of energy synthesis, MQC imbalances emerge as critical contributors to diabetic encephalopathy (DE) and peripheral nerve damage, disrupting cellular resilience and metabolic homeostasis [193].

Impaired mitogenesis underpins cognitive deficits in diabetes. Phosphorylation of WW domain-containing oxidoreductase 1 (WWOX) at tyrosine 33 under hyperglycemia exacerbates mitogenesis defects and ROS overproduction, triggering neuronal apoptosis in neuroblastoma models [194]. Conversely, activation of dopamine D1 receptors (DRD1) rescues AMPK/PGC-1α signaling, restoring mitochondrial biogenesis and alleviating cognitive dysfunction in diabetic mice [195]. Similarly, sodium butyrate (NaB), a gut-derived metabolite, enhances hippocampal synaptic plasticity by reviving AMPK/PGC-1α-driven mitogenesis, highlighting metabolic-epigenetic crosstalk in T2DM neuroprotection [196].

Dysregulated mitochondrial dynamics further propagate neuronal injury. Diabetic models reveal diminished OPA1 and MFN1/2 alongside elevated Drp1 and Fis1, reflecting fission-fusion imbalance in hippocampal and peripheral neurons [197]. Lipin1 deficiency, by altering mitochondrial membrane phospholipids, disrupts synaptic mitochondrial dynamics, exacerbating cognitive decline [197]. Caveolin-1 (Cav-1) counters these effects by suppressing GSK3β/Drp1-mediated fission and enhancing AMPK/PINK1/Parkin- and ULK1-dependent mitophagy, though excessive Drp1 inhibition may paradoxically worsen neurodegeneration [198, 199]. In peripheral nerves, mitochondrial calcium uniporter (MCU) deletion mitigates neuropathic pain by curbing calcium-dependent fission, preserving small-fiber integrity in diabetic mice [200].

Mitophagy dysfunction amplifies diabetic neuropathology. Hyperglycemia and advanced glycation end-products (AGEs) impair hippocampal mitophagy via Keap1/Nrf2/PHB2 suppression, accelerating cognitive decline [201]. Brain-derived neurotrophic factor (BDNF) counteracts oxidative stress by activating TrkB/HIF-1α/BNIP3 signaling, restoring mitophagy in cerebral microvasculature [202]. NaB further rescues neuronal mitophagy by blocking RELA-HDAC8 repression of Parkin, while the SIRT1 activator piceatannol (PCN) enhances both mitogenesis and mitophagy via SIRT1/PGC1α and SIRT1/PINK1/Parkin axes, alleviating peripheral neuropathy [203, 204]. SIRT3 overexpression attenuates painful DPN through FoxO3a/PINK1/Parkin-mediated mitophagy, whereas PARP1 hyperactivity drives peripheral nerve injury via mitophagy suppression [205, 206]. These mechanisms underscore mitochondrial dysregulation as a nexus of diabetic neuropathies, with therapeutic strategies targeting MQC offering promise to preserve neuronal integrity and function.

**Fig. 2:**
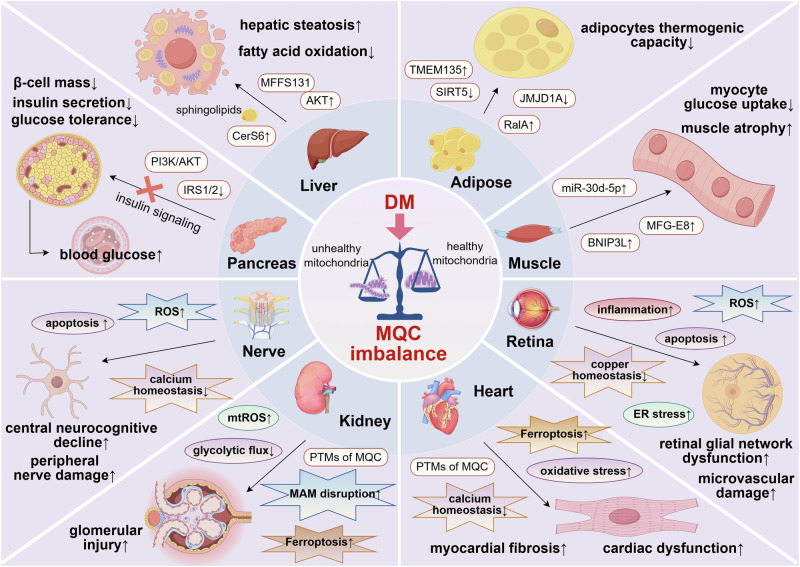
MQC imbalance in the pathophysiology of diabetes and its complications (Created with FigDraw). **Pancreas:** MQC failure in islets impairs β-cell mass, insulin secretion and glucose tolerance by harming insulin signaling, which excerbates insulin resistance and elevates blood glucose. **Liver:** Hepatic MQC imbalance induces hepatic steatosis and fatty acid oxidation. **Adipose:** Adipocyte MQC imbalance decreases adipocytes thermogenic capacity and influences systemic energy balance. **Muscle:** MQC failure impairs insulin-stimulated myocyte glucose uptake and promotes muscle atrophy. **Retina:** MQC damage induces retinal glial network dysfunction and microvascular damage. **Heart:** The myocardial fibrosis and cardiac dysfunction in DCM are aggravated by an interplay of MQC defects, ferrotopsis and oxidation stress. **Kidney**: MQC dysfunction is contributed to glomerular injury in diabetic nephropathy through promoting mitochondrial ROS, MAM dysruption and ferrotopsis. **Nerve:** MQC imbalance induce both central neurocognitive decline and peripheral nerve damage by promoting apoptosis, ROS and calcium metabolism disorder.

Overall, MQC imbalance plays an important role in the pathophysiology of diabetes and diabetic complications (Fig. 2).

## Therapeutic potentials for diabetic complications by targeting MQC

Current therapeutic paradigms for diabetic vascular complications, often siloed by organ-specific pathology, inadequately address the systemic MQC dysfunction underpinning these conditions. MQC has emerged as a unifying therapeutic axis across diabetic angiopathies. Aligning with clinical guidelines, repurposing existing therapies and exploring natural compounds to modulate MQC pathways offers transformative potential for multisystem vascular protection.

### Clinically approved hypoglycemic agents

Several glucose-lowering agents exert their pleiotropic effect on diabetic complications by targeting MQC (Table 5). Metformin, an AMPK activator, efficiently prevents high glucose induced mitochondrial ultrastructural and functional abnormalities in human islet cells [207]. It has beneficial clinical implications on promoting mitochondrial function and mitophagy in type 2 diabetic patients, which alleviates retinopathy and nephropathy by restoring AMPK-dependent MQC balance and inhibiting Drp1-mediated fission in atherosclerosis models. It further rescues cognitive deficits via PGC-1α-driven mitogenesis and enhances post-stroke recovery through mitophagy activation [208–213]. DPP-4 inhibitors demonstrate class-wide vascular benefits. Alogliptin and evogliptin enhance cardiac mitogenesis via PGC-1α/NRF1/TFAM signaling in DCM, while sitagliptin restores renal mitochondrial dynamics through SDF-1α/CXCR4/STAT3. Vildagliptin attenuates endothelial fission via AMPK/Drp1 inhibition and reduces arrhythmia risk by promoting mitogenesis [214–219]. SGLT2 inhibitors, including empagliflozin and canagliflozin, mitigate renal and cardiac injury by suppressing fission and enhancing mitophagy. Empagliflozin alleviates tubulopathy and myocardial microvascular damage, whereas canagliflozin activates PINK1/Parkin-mediated mitophagy in DCM [220–224]. GLP-1 receptor agonists exhibit tissue-specific modulation. Exendin-4 reverses cardiac mitochondrial abnormalities and curbs vascular calcification via AMPK-dependent mitophagy, while liraglutide paradoxically suppresses PINK1/Parkin signaling to protect endothelial function [225–227]. Pioglitazone and Exendin-4 exert neuroprotective effects by targeting altered brain mitogenesis in T1DM [228].

**Table 5:** Summary of MQC-targeted clinical drugs in anti-diabetic complications.

Clinical drugs		Diabetic Vascular complications	Molecule targets	MQC mechanisms	Reference
Metformin		Diabetic retinopathy	AMPK, MFN1, PGC-1α, TFAM	Inhibiting fission, promoting mitogenesis, and mitophagy	[208]
		Renal oxidative stress and tubulointerstitial fibrosis	AMPK, PINK1, PARKIN	Promoting mitophagy	[209]
		Human renal epithelial cell injury	PP2A, NF-κB, PARKIN	Promoting mitophagy	[210]
		Diabetes-accelerated atherosclerosis	AMPK, DRP1	Inhibiting fission	[211]
		HG-aggravated cerebral ischemia/reperfusion injury	AMPK, ULK1, PINK1, PARKIN	Promoting mitophagy	[213]
DPP-4 inhibitors	Alogliptin	Diabetic diastolic dysfunction and Atrial Remodeling	AMPK, PGC-1α, NRF1, TFAM	Promoting mitogenesis	[214, 215]
	Sitagliptin	Renal tubular injury	SDF-1α, CXCR4, STAT3	Inhibiting fission	[216]
	Vildagliptin	Endothelial dysfunction	AMPK, Drp1	Inhibiting fission	[217]
		Diabetic myocardial ischemia-induced arrhythmogenesis	PGC-1α, SIRT1, NRF-2	Inhibiting mitogenesis	[218]
	evogliptin	Diabetic cardiomyopathy	PGC1α, NRF1, TFAM	Inhibiting mitogenesis	[219]
SGLT-2 inhibitors	Empagliflozin	Diabetic renal tubular injury	AMPK, SP1, PGAM5	Inhibiting fission	[220]
		Diabetic tubulopathy	DRP1, MFN1	Inhibiting fission	[221]
		Myocardial microvascular injury	AMPK, DRP1	Inhibiting fission	[222]
		Diabetic cardiomyopathy	AMPK, PGC-1α	Inhibiting mitogenesis	[223]
	Canagliflozin	Diabetic cardiomyopathy	PINK1, PARKIN	Inhibiting mitophagy	[224]
GLP-1 receptor agonists	Exendin-4	Diabetic cardiomyopathy	MFN2, DRP1, FIS1, PARKIN	Inhibiting fission and mitophagy	[225]
		Vascular calcification	AMPK	Inhibiting mitophagy	[226]
	Liraglutide	Endothelial dysfunction	PINK1, PARKIN	Inhibiting mitophagy	[227]

*DPP-4* dipeptidyl peptidase-4；*SGLT-2* sodium-glucose cotransporter-2, *GLP-1* glucagon-like peptide-1.

### Natural products and herbal compounds

Natural products, rich in bioactive phytochemicals, offer diverse mechanisms to restore MQC (Table 6). Rosmarinic acid and resveratrol attenuate cardiac dysfunction through SIRT1-mediated PGC-1α deacetylation, while *Astragalus*-derived astragaloside IV enhances mitogenesis via SIRT1/PGC1α/NRF1 and stabilizes mitochondrial dynamics in nephropathy [229–233]. Salidroside and astragalin activate AMPK/SIRT3-PGC-1α axes, mitigating renal and myocardial fibrosis [234–236]. Rhein and Gynostemma pentaphyllum combat retinal and cardiac injury via AMPK/NRF2 signaling, whereas berberine restores podocyte homeostasis by balancing PGC-1α-mediated biogenesis and Drp1 inhibition [237–243]. Traditional formulas like Jinlida granules and Si-Miao-Yong-An decoction improve renal and cardiac outcomes through AMPK/PGC-1α activation and PPARα modulation [244, 245].

**Table 6:** Summary of MQC-targeted natural products in anti-diabetic complications.

Natural products	Source	Chemical structure	Molecular formula	Complica-tions	In vivo	In vitro	Targets	MQC Mechanisms	Reference
Resveratrol	*Rhizoma Polygoni Cuspidati*		C_14_H_12_O_3_	DCM	HFD/STZ-induced rats	H9c2 cells with HG	SIRT1/PGC-1α	Promoting mitogenesis	[229]
Rosmarinic acid	*Rosemary*		C_18_H_16_O_8_	DCM	HFD/STZ-induced mice	H9c2 cells with HG	SIRT1/PGC-1α	Promoting mitogenesis	[230]
Astragaloside IV	*Astragalus membranaceus (Fisch.) Bge*.		C_41_H_68_O_14_	DKD	Db/db mice	Mouse podocytes with phenylsulfate	SIRT1/PGC1α /NRF1	Promoting mitogenesis	[231]
					Db/db mice	/	DRP1,FIS1,PINK1,PARKIN	Inhibiting fission and mitophagy	[218]
Astragaloside II	*Astragalus membranaceus (Fisch.) Bge*.		C_43_H_70_O_15_	DKD	STZ-induced rats	/	MFN2, FIS1,PINK1, PARKIN	Inhibiting fission, promoting fusion and mitophagy	[233]
Salidroside	*Rhodiola rosea*		C_14_H_20_O_7_	DKD	HFD/STZ-induced mice	/	SIRT1/PGC-1α	Promoting mitogenesis	[234]
				DCM	HFD/STZ-induced mice	/	SIRT3/AMPK; PGC-1α/TFAM	Promoting mitogenesis	[235]
Astragalin	*Thesium Chinense Turcz*		C_21_H_20_O_11_	DKD	STZ-induced mice	HK-2 cells with HG and PA	AMPK/PGC-1α	Promoting mitogenesis	[236]
Rhein	*Rheum palmatum L*.		C_15_H_8_O_6_	DR	/	Müller cells with HG	AMPK/SIRT1/PGC-1α	Promoting mitogenesis	[237]
Gynostemma pentaphyllum	*Gynostemma pentaphyllum (Thunb.) Makino*		C_41_H_68_O_13_	DCM	HFD/STZ-induced rats	/	AMPK/NRF2/HO-1	Promoting mitogenesis	[238]
Icariin	*Epimedium*		C_33_H_40_O_15_	DCM	Db/db mice	NMCMs with HG	Apelin/Sirt3	Promoting mitogenesis and fusion	[239]
				DKD	STZ-induced rats	MPC-5 cells with HG	Sesn2	Promoting mitophagy	[241]
Berberine	*Berberis vulgaris;Coptis chinensis*		C_20_H_18_NO_4_^+^	DKD	Db/db mice	Mouse podocytes with PA	PGC-1α	Promoting mitogenesis	[242]
					Db/db mice	Mouse podocytes with PA	DRP1	Inhibiting fission	[243]
Jinlida granules	*/*	/	/	DKD	Db/db mice	MPC-5 cells with HG	AMPK/PGC-1α	Promoting mitogenesis	[244]
Si-Miao-Yong-An decoction	*/*	/	/	DCM	STZ-induced mice	/	GLC/PPARα/PGC-1α	Promoting mitogenesis	[245]
Tanshinone IIa	*Salvia miltiorrhiza Bunge*		C_19_H_18_O_3_	DR	/	BRECs with MGO	GLO1	Inhibiting fission	[181]
Supplemented Gegen Qinlian Decoction	*/*	/	/	DKD	HFD/STZ-induced rats	MPC-5 cells with HG	RIPK1/RIPK3/MLKL	Inhibiting fission	[246]
Paeonol	*Paeonia albiflora*		C_9_H_10_O_3_	DCM	STZ-induced rats	NRCMs with HG	CK2α/JAK2/STAT3/OPA1	Promoting fusion	[247]
Punicalagin	*Punica granatum L*.		C_48_H_28_O_30_	DCM	STZ-induced rats	NRCMs with HG	PTP1B/STAT3/OPA1	Promoting fusion	[248]
Modified Hu-lu-ba-wan	*/*	/	/	DKD	Db/db mice	MPC-5 cells with AGEs	PKM2/PGC-1α/OPA1	Promoting fusion, inhibiting fission	[249]
Notoginsenoside Fc	*P.notoginseng*		C_58_H_98_O_26_	DKD	Db/db mice	MGECs with HG	HMGCS2	Promoting fusion, inhibiting fission	[250]
Asiatic acid	*Centella asiatica*		C_30_H_48_O_5_	DKD	STZ-induced rats	HK-2 cells with AGEs	NRF-2	Promoting fusion, inhibiting fission	[251]
Fufang Zhenzhu Tiaozhi	*/*	/	/	DCM	HFD/STZ-induced mice	H9c2 cells with PA	MFN2, OPA1,DRP1, FIS1	Promoting fusion, inhibiting fission	[252]
Dioscin	*Dioscorea zingiberensis C.H. Wright*		C_45_H_72_O_16_	DKD	HFD/STZ-induced rats	/	MFN2,DRP1,PINK1,PARKIN	Promoting fusion and mitophagy, inhibiting fission	[253]
Baicalin	*Scutellaria baicalensis*		C_21_H_18_O_11_	DCM	Db/db mice	H9c2 cells with HG	SENP1/SIRT3	Promoting fusion and mitophagy, inhibiting fission	[254]
Notoginsenoside R1	*Panax notoginseng*		C_47_H_80_O_18_	DR	Db/db mice	rMC-1 cells with HG	PINK1/PARKIN	Promoting mitophagy	[255]
Germacrone	*Curcuma kwangsiensis S.G.Lee et C.F.Liang*		C_15_H_22_O	DKD	Db/db mice	HK-2 cells with HG	PINK1/PARKIN	Promoting mitophagy	[256]
Huangqi-Danshen decoction	*/*	/	/	DKD	Db/db mice	/	PINK1/PARKIN	Promoting mitophagy	[257]
Qing-Re-Xiao-Zheng-Yi-Qi Formula	*/*	/	/	DKD	STZ-induced rats	MPC-5 cells with HG	PINK1/PARKIN	Promoting mitophagy	[258]
San-Huang-Yi-Shen Capsule	*/*	/	/	DKD	HFD/STZ-induced rats	/	PINK1/PARKIN	Promoting mitophagy	[259]
JinChanYiShen TongLuo Formula	*/*	/	/	DKD	STZ-induced rats	HK-2 cells with HG	HIF-1α/PINK1/PARKIN	Promoting mitophagy	[260]
Huangkui capsule	*/*	/	/	DKD	HFD/STZ-induced mice	HK-2 cells with HG	STING1/PINK1	Promoting mitophagy	[261]
Hyperforin	*Hypericum perforatum L*.		C_35_H_52_O_4_	DKD	HFD/STZ-induced mice; db/db mice	HK-2 cells with HG	DLAT/AMPK	Promoting mitophagy	[171]
Ginsenoside Ro	*Panax ginseng C. A. Meyer*		C_48_H_76_O_19_	DR	HFD/STZ-induced mice	hRMECs with AGEs	EPAC1/AMPK	Promoting mitophagy	[262]
Poricoic acid A	*Poria cocos (Schw.) Wolf*		C_31_H_46_O_5_	DKD	/	MPC-5 cells with HG	FUNDC1	Promoting mitophagy	[263]
Heyingwuzi formulation	*/*	/	/	DR	STZ-induced mice	HRCECs with HG	HIF-1α/BNIP3/NIX	Promoting mitophagy	[264]

Mitochondrial dynamics are further regulated by compounds such as tanshinone IIa, which activates GLO1 to counteract retinal fission, and paeonol, which enhances OPA1 via CK2α/JAK2-STAT3 signaling in DCM [181, 246–248]. Modified Hu-lu-ba-wan and notoginsenoside Fc restore glomerular integrity by modulating PKM2/PGC-1α/OPA1 and HMGCS2-mediated dynamics [249, 250].

Natural products also alleviate diabetic complications by targeting mitophagy [251–254]. Notoginsenoside R1 and germacrone enhance PINK1-dependent pathways in retinopathy and nephropathy, while Huangqi-Danshen decoction and hyperforin activate STING1/PINK1 or DLAT-AMPK axes to bolster autophagic flux [255–261]. Ginsenoside Ro, a *Panax ginseng*-derived compound, preserves retinal endothelial integrity by restoring EPAC1/AMPK-mediated mitophagy, countering diabetic retinopathy [262]. Poricoic acid A, sourced from *Poria cocos*, mitigates podocyte injury via FUNDC1 activation, a receptor-dependent mitophagy pathway critical for renal protection [263]. Heyingwuzi formulation, a traditional Chinese medicine, alleviates retinal damage by suppressing ROS and apoptosis through HIF-1α/BNIP3/NIX-driven mitophagy, highlighting alternative pathways for vascular rescue [264].

### Emerging therapeutic strategies

Beyond conventional and natural therapies, exogenous agents like melatonin restore cardiac MQC and myocardial ischemia/reperfusion injury in T1DM via AMPK/PGC-1αsignaling [265, 266]. Photobiomodulation modulates mitochondrial dynamics in the sciatic nerve and in the dorsal root ganglia neurons, alleviating peripheral nervous system in T1DM [267]. Immunotherapeutic approach anti-CD3 monoclonal antibodies targets exhausted CD8 + T cells’ energy metabolism and suppresses T cell signaling through regulation of Drp1-mediated mitochondrial dynamics in T1DM [268]. Finerenone rescues renal mitophagy through PI3K/Akt/eNOS pathways [269]. Placental mesenchymal stem cells rejuvenate podocyte mitophagy via SIRT1/PGC-1α/TFAM, highlighting regenerative medicine’s potential [270].

In a word, these findings underscore MQC as a nexus for therapeutic innovation in diabetic angiopathy. While repurposed drugs and natural compounds show promise, challenges remain in optimizing tissue-specific targeting, resolving mechanistic contradictions, and advancing combinatorial approaches. Translational validation and clinical trials are imperative to harness MQC modulation for multisystem vascular protection.

## Conclusions

This review synthesizes emerging insights into MQC as a central axis in the pathophysiology of DM and its vascular complications, emphasizing the interplay between mitochondrial dysfunction and metabolic dysregulation. Mitochondrial anomalies drive oxidative stress, inflammation, and cellular demise, positioning MQC as a pivotal therapeutic frontier. Despite advances in understanding MQC mechanisms, translational gaps persist, particularly in targeting diabetic complications, which remain understudied compared to primary metabolic defects. Current therapies, including clinical hypoglycemic agents and natural compounds, demonstrate pleiotropic benefits by restoring mitochondrial homeostasis, yet lack specificity for vascular pathologies. Natural products, with their multi-target capacity to modulate MQC pathways, offer promising scaffolds for drug development but require rigorous clinical validation to address challenges such as bioavailability and tissue selectivity. Furthermore, although majority of the therapeutic drugs have proved to be clinical effective, the reported data related to MQC mostly come from experiments with rodent and cell models, while studies conducted in humans (ex vivo and in vivo) are still relatively few and lack higher quality evidence, which provides further direction for our future research. Future research must prioritize combinatorial strategies, precision targeting of MQC nodes, and mechanistic elucidation of organ-specific mitochondrial crosstalk. By bridging molecular insights with therapeutic innovation, this review underscores the potential of MQC-centric approaches to redefine the management of diabetic angiopathies, urging interdisciplinary efforts to transform preclinical promise into clinical reality.
