# Correction: Mitochondrial quality control in diabetes mellitus and complications: molecular mechanisms and therapeutic strategies

**DOI:** 10.1038/s41419-025-08078-x

**Published:** 2025-12-23

**Authors:** Yanling Chen, Xun Liu, Yixuan Liu, Yujia Li, Dingxiang Li, Zhigang Mei, Yihui Deng

**Affiliations:** 1 School of Integrated Chinese and Western Medicine, Hunan University of Chinese Medicine, Changsha, China; 2 Key Laboratory of Hunan Province for Integrated Traditional Chinese and Western Medicine on Prevention and Treatment of Cardio-Cerebral Diseases, Hunan University of Chinese Medicine, Changsha, China; 3 Loudi Central Hospital, Loudi, China; 4 School of Traditional Chinese Medicine, Hunan University of Chinese Medicine, Changsha, China

**Keywords:** Diabetes, Molecular biology

Correction to: *Cell Death & Disease* (2025) **16**:652 10.1038/s41419-025-07936-y, published online 27 August 2025

During the production process, Table 6 has been typeset erroneously.

**Table 6:** Summary of MQC-targeted natural products in anti-diabetic complications.

Natural Products	Source	Chemical Structure	Molecular Formula	Complica-tions	In vivo	In vitro	Targets	MQC Mechanisms	Reference
Resveratrol	*Rhizoma Polygoni Cuspidati*		C_14_H_12_O_3_	DCM	HFD/STZ-induced rats	H9c2 cells with HG	SIRT1/PGC-1α	Promoting mitogenesis	[229]
Rosmarinic acid	*Rosemary*		C_18_H_16_O_8_	DCM	HFD/STZ-induced mice	H9c2 cells with HG	SIRT1/PGC-1α	Promoting mitogenesis	[230]
Astragaloside IV	*Astragalus membranaceus (Fisch.) Bge*.		C_41_H_68_O_14_	DKD	Db/db mice	Mouse podocytes with phenylsulfate	SIRT1/PGC1α /NRF1	Promoting mitogenesis	[231]
					Db/db mice	/	DRP1,FIS1,PINK1,PARKIN	Inhibiting fission and mitophagy	[218]
Astragaloside II	*Astragalus membranaceus (Fisch.) Bge*.		C_43_H_70_O_15_	DKD	STZ-induced rats	/	MFN2, FIS1,PINK1, PARKIN	Inhibiting fission, promoting fusion and mitophagy	[233]
Salidroside	*Rhodiola rosea*		C_14_H_20_O_7_	DKD	HFD/STZ-induced mice	/	SIRT1/PGC-1α	Promoting mitogenesis	[234]
				DCM	HFD/STZ-induced mice	/	SIRT3/AMPK; PGC-1α/TFAM	Promoting mitogenesis	[235]
Astragalin	*Thesium Chinense Turcz*		C_21_H_20_O_11_	DKD	STZ-induced mice	HK-2 cells with HG and PA	AMPK/PGC-1α	Promoting mitogenesis	[236]
Rhein	*Rheum palmatum L*.		C_15_H_8_O_6_	DR	/	Müller cells with HG	AMPK/SIRT1/PGC-1α	Promoting mitogenesis	[237]
Gynostemma pentaphyllum	*Gynostemma pentaphyllum (Thunb.) Makino*		C_41_H_68_O_13_	DCM	HFD/STZ-induced rats	/	AMPK/NRF2/HO-1	Promoting mitogenesis	[238]
Icariin	*Epimedium*		C_33_H_40_O_15_	DCM	Db/db mice	NMCMs with HG	Apelin/Sirt3	Promoting mitogenesis and fusion	[239]
				DKD	STZ-induced rats	MPC-5 cells with HG	Sesn2	Promoting mitophagy	[241]
Berberine	*Berberis vulgaris;Coptis chinensis*		C_20_H_18_NO_4_^+^	DKD	Db/db mice	Mouse podocytes with PA	PGC-1α	Promoting mitogenesis	[242]
					Db/db mice	Mouse podocytes with PA	DRP1	Inhibiting fission	[243]
Jinlida granules	*/*	/	/	DKD	Db/db mice	MPC-5 cells with HG	AMPK/PGC-1α	Promoting mitogenesis	[244]
Si-Miao-Yong-An decoction	*/*	/	/	DCM	STZ-induced mice	/	GLC/PPARα/PGC-1α	Promoting mitogenesis	[245]
Tanshinone IIa	*Salvia miltiorrhiza Bunge*		C_19_H_18_O_3_	DR	/	BRECs with MGO	GLO1	Inhibiting fission	[181]
Supplemented Gegen Qinlian Decoction	*/*	/	/	DKD	HFD/STZ-induced rats	MPC-5 cells with HG	RIPK1/RIPK3/MLKL	Inhibiting fission	[246]
Paeonol	*Paeonia albiflora*		C_9_H_10_O_3_	DCM	STZ-induced rats	NRCMs with HG	CK2α/JAK2/STAT3/OPA1	Promoting fusion	[247]
Punicalagin	*Punica granatum L*.		C_48_H_28_O_30_	DCM	STZ-induced rats	NRCMs with HG	PTP1B/STAT3/OPA1	Promoting fusion	[248]
Modified Hu-lu-ba-wan	*/*	/	/	DKD	Db/db mice	MPC-5 cells with AGEs	PKM2/PGC-1α/OPA1	Promoting fusion, inhibiting fission	[249]
Notoginsenoside Fc	*P.notoginseng*		C_58_H_98_O_26_	DKD	Db/db mice	MGECs with HG	HMGCS2	Promoting fusion, inhibiting fission	[250]
Asiatic acid	*Centella asiatica*		C_30_H_48_O_5_	DKD	STZ-induced rats	HK-2 cells with AGEs	NRF-2	Promoting fusion, inhibiting fission	[251]
Fufang Zhenzhu Tiaozhi	*/*	/	/	DCM	HFD/STZ-induced mice	H9c2 cells with PA	MFN2, OPA1,DRP1, FIS1	Promoting fusion, inhibiting fission	[252]
Dioscin	*Dioscorea zingiberensis C.H. Wright*		C_45_H_72_O_16_	DKD	HFD/STZ-induced rats	/	MFN2,DRP1,PINK1,PARKIN	Promoting fusion and mitophagy, inhibiting fission	[253]
Baicalin	*Scutellaria baicalensis*		C_21_H_18_O_11_	DCM	Db/db mice	H9c2 cells with HG	SENP1/SIRT3	Promoting fusion and mitophagy, inhibiting fission	[254]
Notoginsenoside R1	*Panax notoginseng*		C_47_H_80_O_18_	DR	Db/db mice	rMC-1 cells with HG	PINK1/PARKIN	Promoting mitophagy	[255]
Germacrone	*Curcuma kwangsiensis S.G.Lee et C.F.Liang*		C_15_H_22_O	DKD	Db/db mice	HK-2 cells with HG	PINK1/PARKIN	Promoting mitophagy	[256]
Huangqi-Danshen decoction	*/*	/	/	DKD	Db/db mice	/	PINK1/PARKIN	Promoting mitophagy	[257]
Qing-Re-Xiao-Zheng-Yi-Qi Formula	*/*	/	/	DKD	STZ-induced rats	MPC-5 cells with HG	PINK1/PARKIN	Promoting mitophagy	[258]
San-Huang-Yi-Shen Capsule	*/*	/	/	DKD	HFD/STZ-induced rats	/	PINK1/PARKIN	Promoting mitophagy	[259]
JinChanYiShen TongLuo Formula	*/*	/	/	DKD	STZ-induced rats	HK-2 cells with HG	HIF-1α/PINK1/PARKIN	Promoting mitophagy	[260]
Huangkui capsule	*/*	/	/	DKD	HFD/STZ-induced mice	HK-2 cells with HG	STING1/PINK1	Promoting mitophagy	[261]
Hyperforin	*Hypericum perforatum L*.		C_35_H_52_O_4_	DKD	HFD/STZ-induced mice; db/db mice	HK-2 cells with HG	DLAT/AMPK	Promoting mitophagy	[171]
Ginsenoside Ro	*Panax ginseng C. A. Meyer*		C_48_H_76_O_19_	DR	HFD/STZ-induced mice	hRMECs with AGEs	EPAC1/AMPK	Promoting mitophagy	[262]
Poricoic acid A	*Poria cocos (Schw.) Wolf*		C_31_H_46_O_5_	DKD	/	MPC-5 cells with HG	FUNDC1	Promoting mitophagy	[263]
Heyingwuzi formulation	*/*	/	/	DR	STZ-induced mice	HRCECs with HG	HIF-1α/BNIP3/NIX	Promoting mitophagy	[264]

The original article has been corrected.

